# Sialic Acids in Kidney Disease: Immune Regulation, Complement Activation and Glomerular Injury

**DOI:** 10.3390/antiox15050626

**Published:** 2026-05-14

**Authors:** Agnese Spennacchio, Gianluca Caridi, Carolina Bigatti, Gabriele Gaggero, Katia Mazzocco, Maria Teresa Gambaudo, Roberta Musso, Valerio Gaetano Vellone, Andrea Angeletti, Xhuliana Kajana

**Affiliations:** 1 Nephrology, Dialysis and Transplantation, IRCCS Istituto Giannina Gaslini, 16147 Genoa, Italy; agnesespennacchio@gaslini.org (A.S.); gianlucacaridi@gaslini.org (G.C.); carolina.bigatti.cb@gmail.com (C.B.); xhulianakajana@gaslini.org (X.K.); 2 Pathology Unit, IRCCS Istituto Giannina Gaslini, 16147 Genoa, Italy; gabrielegaggero@gaslini.org (G.G.); katiamazzocco@gaslini.org (K.M.); mariateresagambaudo@gaslini.org (M.T.G.); robertamusso@gaslini.org (R.M.); valeriovellone@gaslini.org (V.G.V.); 3 Department of Integrated Surgical and Diagnostic Sciences (DISC), University of Genoa, 16126 Genoa, Italy

**Keywords:** sialic acids, sialylation, kidney disease, complement system, podocyte

## Abstract

Oxidative stress and inflammation are key drivers of kidney injury and disease progression. In this context, the role of sialic acids emerged as a critical regulatory layer linking redox imbalance, immune activation, and tissue damage. Sialic acids are terminal negatively charged residues that regulate complement activity, immune cell signaling, and the structural integrity of the glomerular filtration barrier. Alterations in sialylation, resulting from impaired biosynthesis or increased sialidase activity, disrupt immune homeostasis, enhance inflammatory responses, and promote complement-mediated injury. In the kidney, these mechanisms contribute to podocyte dysfunction, glomerular inflammation, and fibrosis and are implicated in glomerulopathies, transplantation, and plasma cell dyscrasias. Emerging evidence also highlights the therapeutic potential of targeting sialic acid metabolism through inhibition of desialylation or restoration of sialylation pathways. Overall, sialic acids represent dynamic modulators at the intersection of oxidative stress and immunity, offering novel opportunities for biomarker development and mechanism-based therapies in kidney disease.

## 1. Introduction

Oxidative stress and inflammation are closely interconnected processes involved in kidney injury and the development of chronic renal damage [1]. Due to its high metabolic demand and extensive vascularization, the kidney is particularly vulnerable to redox imbalance, where disruption of the balance between reactive oxygen species (ROS) and antioxidant defenses leads to cellular damage and altered renal hemodynamics [2,3,4].

Within this pathogenic framework, glycobiology has emerged as an additional and highly relevant regulatory layer. Terminal sialic acid residues on glycoproteins and glycolipids play a pivotal role in maintaining renal structural integrity and immune homeostasis [4,5]. Located at the cell surface and characterized by negative charge, sialic acids contribute to the stability of the glomerular filtration barrier and the endothelial glycocalyx, protecting against mechanical stress, oxidative injury, and inappropriate immune activation [5].

Increasing evidence suggests that dysregulation in sialic acid metabolism, resulting from impaired synthesis or enhanced sialidase activity, is associated with several pathological conditions characterized by systemic and tissue inflammation [5,6].

Among the proposed mechanisms, enzymatic desialylation may enhance alloimmune responses by exposing underlying glycan structures, modifying ligand–receptor interactions, and increasing susceptibility to complement activation and immune-mediated injury, thereby promoting inflammatory responses [5].

**Figure 1:**
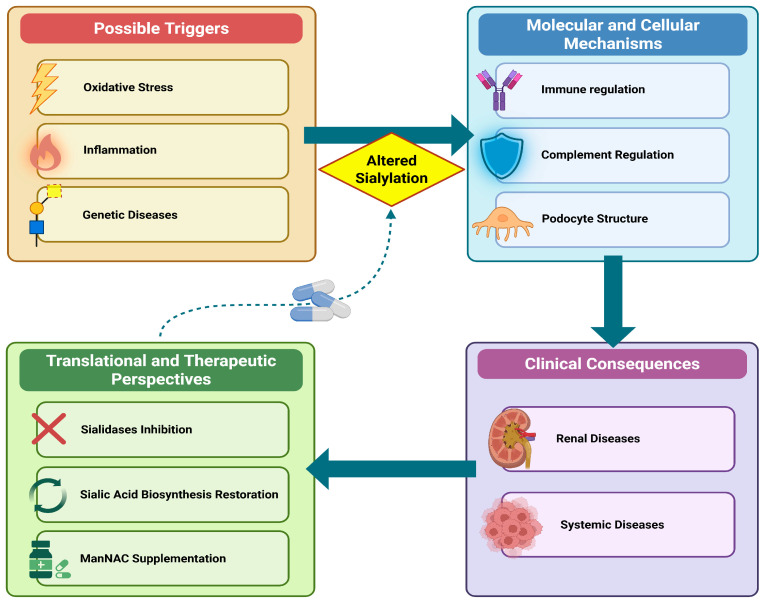
Visual summary. Graphical overview of the triggers, molecular mechanisms, clinical consequences, and therapeutic perspectives associated with altered sialylation.

This review aims to examine the emerging role of sialic acids in kidney disease, with particular emphasis on pathological contexts characterized by immune dysregulation, oxidative stress, and chronic inflammation. We focus on inflammatory conditions where alterations in sialylation may directly affect glomerular integrity, immune regulation, and systemic inflammatory responses. In this framework, sialic acids are proposed as dynamic regulators at the interface between oxidative stress–induced tissue injury and immune activation, potentially acting as previously underappreciated modulators of renal disease progression (Figure 1). This narrative review was conducted in accordance with the SANRA (Scale for the Assessment of Narrative Review Articles) guidelines: literature was retrieved from PubMed, Google Scholar, and Web of Science using keywords related to sialic acid, neuraminidase, sialylation, complement system, oxidative stress, inflammation, podocyte biology, and kidney disease. Original articles, reviews, and experimental studies were selected based on their relevance, methodological quality, and contribution to the understanding of altered sialylation in kidney disease. Additional studies were identified through manual screening of reference lists. Particular attention was given to recent and impactful evidence while also including landmark earlier studies to provide historical and mechanistic context. However, due to accessibility constraints, non-English publications and some gray literature may not have been fully included.

## 2. Biological Background

### 2.1. Sialic Acid in Glycobiology

Sialic acids comprise a family of structurally related monosaccharides that occupy terminal positions on glycoconjugates and exert a major influence on molecular recognition and biological function. In humans, N-acetylneuraminic acid (Neu5Ac) represents the predominant form. In contrast, N-glycolylneuraminic acid (Neu5Gc) is largely absent in humans [4,5], although it may be incorporated from dietary sources and has been implicated in inflammatory and immune-mediated processes [5,6]. The expression and turnover of sialic acids are tightly regulated by the coordinated activity of sialyltransferases, which catalyze their addition through specific glycosidic linkages (α2–3, α2–6, or α2–8), and sialidases (neuraminidases), which mediate their removal from glycoconjugates [5,7,8].

The terminal positioning and negative charge of sialic acids critically influence the physicochemical and functional properties of glycoconjugates. Sialylated glycans regulate cell–cell interactions, modulate protein conformation, and affect the stability and half-life of both circulating and membrane-associated glycoproteins [8,9]. Sialylation also plays a key role in the regulation of the complement cascade, coagulation pathways and immune recognition. Consistently, the selective enzymatic removal of sialic acids by purified sialidases has demonstrated their functional relevance in these processes [5,6].

Beyond their structural roles, sialic acids function as self-associated molecular patterns that actively contribute to immune tolerance. By engaging sialic acid-binding immunoglobulin-like lectins (SIGLECs) expressed on innate immune cells, sialylated glycans transmit inhibitory signals that restrain immune activation and limit excessive inflammation. Accordingly, the integrity of cell surface sialylation is essential for maintaining immunological homeostasis. Loss of sialic acids represents an early and decisive event in glycoconjugate remodeling, leading to exposure of underlying glycan motifs, enhanced complement activation, and increased immune cell engagement [5,7,8].

### 2.2. Sialic Acid as a Biomarker and Modulator of Inflammation and Oxidative Stress

Accumulating evidence indicates that circulating sialic acid levels are directly associated with systemic inflammation and oxidative stress across several diseases, including atherosclerosis, chronic heart failure, and chronic viral hepatitis, and may serve as a surrogate marker of inflammatory burden [10,11,12].

However, sialic acid is not merely a passive marker of inflammation but may actively regulate inflammation and redox pathways. Distinct sialic acid species exert divergent biological effects. Dietary Neu5Gc is associated with enhanced inflammatory responses, whereas Neu5Ac and exogenous sialic acid administration exert anti-inflammatory and antioxidant effects. These include reductions in endotoxemia, oxidative stress and tissue injury [10,12]. Mechanistically, sialic acids act as an active oxygen scavenger and directly detoxify hydrogen peroxide via a stoichiometric reaction that generates stable, non-toxic products, thereby limiting ROS-mediated cytotoxicity and cellular damage [13,14].

Sialylation is a key post-translational modification that regulates both innate and adaptive immunity. Through interactions with sialic acid–binding receptors such as SIGLECs and selectins, sialylated glycans influence immune cell activation, leukocyte trafficking, and inflammatory signaling. Sialylation of immunoglobulins, particularly within the Fc domain, also shapes antibody effector functions in both physiological and pathological contexts [5,15].

Sialic acid regulates inflammatory and oxidative processes through multiple pathways. Sialylation patterns modulate host immunity: IgG glycosylation determines its pro- or anti-inflammatory activity, and specific sialic acid species promote anti-inflammatory cytokine release, such as IL-4 and IL-10 [10]. Sialylated glycans also regulate immune cell activation through interactions with SIGLECs, sialic acid receptors expressed on immune cells. SIGLECs modulate inflammatory responses by regulating signaling downstream of pattern-recognition receptors and by regulating adaptive immunity [10]. In humans, SIGLECs include inhibitory and activating receptors: SIGLEC-2, -3, and -5 to -11 generally attenuate pro-inflammatory signaling, whereas SIGLEC-14, -15, and -16 promote inflammation through MAPK and AKT pathways. Sialylation also controls SIGLEC-mediated antigen handling, including receptor internalization, antigen presentation, and phagocytic recognition [15].

In parallel, microbiota-dependent mechanisms also contribute to these processes. In the gut, sialic acid residues released from host glycans support the expansion of sialic acid–utilizing pathogens, increasing pro-inflammatory cytokine production and sustaining mucosal inflammation. Conversely, exogenous Neu5Gc may promote immune complex formation through interactions with circulating anti-Neu5Gc antibodies, potentially sustaining chronic inflammation [10].

**Figure 2:**
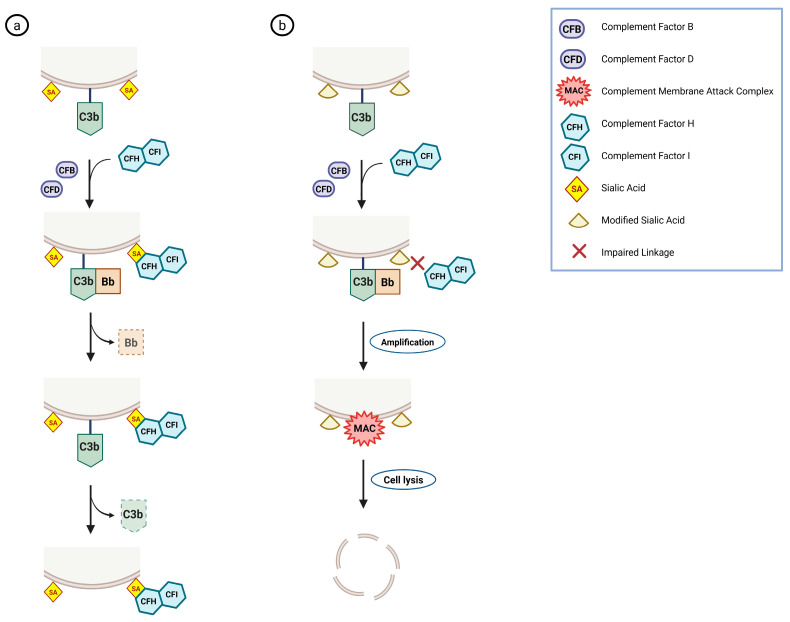
Role of sialic acids in complement regulation. (**a**) On normal cells, surface sialic acids recruit factor H (and factor I), promoting C3bBb dissociation and C3b inactivation, thereby preventing complement-mediated damage. (**b**) On damaged or non-self cells lacking sialylated glycans, the alternative complement pathway remains active, leading to C3bBb amplification, MAC formation, and cell lysis.

As previously mentioned, sialic acids are also involved in regulating the complement system [16,17]. The complement cascade is a central component of innate immunity that facilitates pathogen opsonization, promotes inflammation, and induces cell lysis. Sialic acids serve as key “self-associated molecular patterns” that help discriminate host cells from pathogens during complement activation [5,11,15]. Factor H, the principal inhibitor of the alternative pathway, recognizes sialic acids on cell surfaces, enabling its recruitment and limiting complement activation. This interaction accelerates dissociation of the C3bBb convertase and enhances factor I-mediated cleavage of C3b. Importantly, sialic acid linkage and chemical modifications influence factor H binding. Alterations such as changes in glycosidic linkage or O-acetylation may impair complement regulation and promote inflammation (Figure 2) [5,15,16,17].

**Figure 3:**
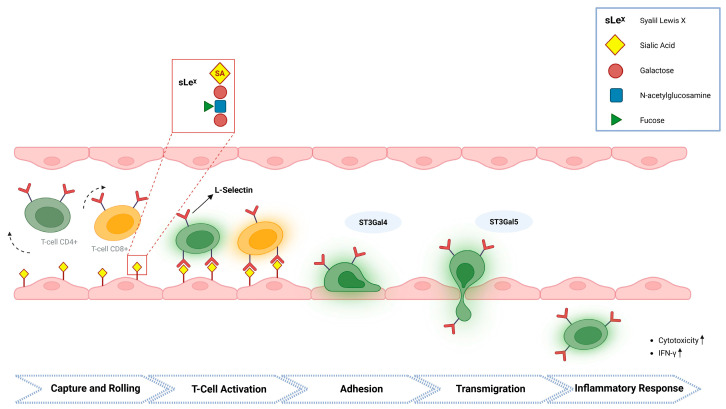
Selectin-mediated CD4+ and CD8+ cell trafficking and sialylated glycans. Leukocyte recruitment to the endothelium occurs through sequential steps including capture and rolling, activation, firm adhesion, and transmigration. Early interactions are mediated by selectins binding to α2,3-sialylated glycans such as sialyl Lewis X (sLe^χ^) expressed on endothelial cells. Glycosyltransferases, including ST3Gal4 and ST3Gal5, regulate the biosynthesis of these ligands and thereby control leukocyte adhesion and migration. Dysregulation of these enzymes impairs immune cell recruitment and inflammatory responses, while proper sialylation enhances T-cell cytotoxicity and IFN-γ production.

Moreover, sialylated glycans influence immune defense through selectin-dependent leukocyte trafficking. Leukocyte recruitment involves capture, rolling, adhesion, and transmigration across the endothelium. Early steps are mediated by selectin interactions with α2,3-sialylated ligands, including sialyl Lewis X (sLe^χ^), expressed on endothelial cells. Glycosyltransferases such as ST3Gal4 and ST3Gal5 regulate these processes, and their dysregulation impairs leukocyte adhesion and inflammatory responses (Figure 3) [15,18,19].

Beyond inflammatory diseases, alterations in sialic acid metabolism are also observed in malignancies, where hypersialylation contributes to immune evasion through inhibitory SIGLECs signaling, reduced complement activation, and impaired macrophage function [14]. Similarly, in cardiovascular disorders, increased circulating sialic acid levels correlate with inflammatory markers and disease progression, likely reflecting vascular injury and systemic inflammatory activation [10,11,14]. In the nervous system, where sialylation is particularly abundant, disruptions in sialoglycan composition and sialidase activity influence neuroinflammation, synaptic function, and neurodegenerative processes [10,11].

Taken together, these observations support a unifying concept: sialic acids act as dynamic regulators of inflammation, oxidative stress, and immune homeostasis. Changes in their expression, distribution, or biochemical modification can reshape immune signaling and cellular interactions across multiple biological systems. Consequently, circulating sialic acid levels and the enzymatic machinery governing sialylation emerge as both informative biomarkers and potential therapeutic targets in diseases characterized by chronic inflammation and redox imbalance.

Building on these mechanisms, alterations in sialylation acquire particular relevance in the kidney, where the integrity of the glomerular filtration barrier and immune-mediated injury are deeply linked.

## 3. Sialic Acid and Kidney

### 3.1. Sialic Acid and the Glomerulus

The glomerulus is a specialized microvascular structure responsible for plasma filtration. It consists of fenestrated endothelial cells, the glomerular basement membrane, and podocytes, which together form the filtration barrier. Glomerular function depends not only on this cellular architecture but also on the biochemical properties of cell surfaces, including the glycocalyx. In the glomerular endothelium, glycosylation is essential for barrier integrity, and disruption of the glycocalyx is associated with albuminuria in diabetes, cardiovascular disease, chronic kidney disease, and experimental models of nephrotic syndrome [20,21,22].

Podocytes form the outermost layer of the filtration barrier. They are specialized epithelial cells with interdigitating foot processes connected by the slit diaphragm. Similar to endothelial cells, podocytes are covered by a dense glycocalyx composed of glycoconjugates, proteoglycans, and glycosaminoglycans, many shared with the glomerular basement membrane. While endothelial glycosylation is well described, the functional relevance of podocyte glycosylation is still far from being completely explored [20,21,22].

**Figure 4:**
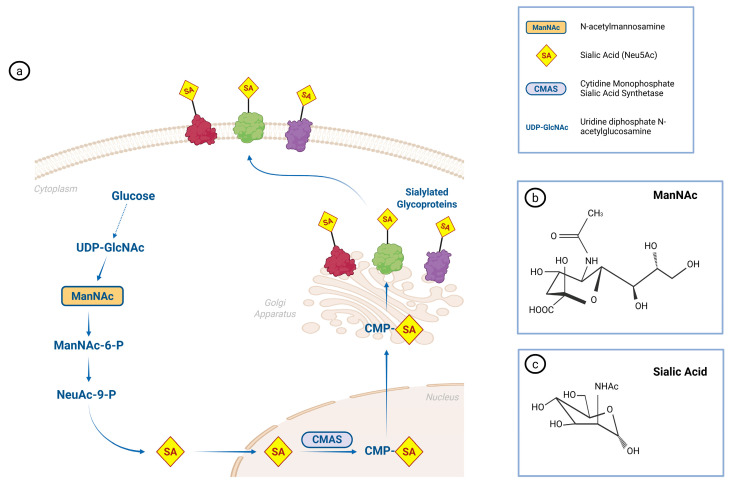
The process of glycoprotein sialylation. (**a**) Biosynthesis pathway of sialic acid (SA, Neu5Ac). Glucose is converted into UDP-GlcNAc, which serves as a precursor for the formation of N-acetylmannosamine (ManNAc), followed by ManNAc-6-phosphate and Neu5Ac-9-phosphate. After dephosphorylation, sialic acid is activated in the nucleus by CMAS to form CMP-Neu5Ac, then transported to the Golgi apparatus, where it is transferred onto glycoproteins, generating sialylated glycoproteins displayed on the cell surface. (**b**) Chemical structure of ManNAc (N-acetylmannosamine). (**c**) Chemical structure of sialic acid.

Sialylation occurs in the Golgi apparatus and depends on the availability of the activated sugar donor CMP–sialic acid, synthesized by CMP–sialic acid synthetase (CMAS) (Figure 4) [20]. A podocyte-specific CMAS knockout mouse model well defines the role of sialylation in podocyte homeostasis. This mouse model selectively ablates podocytes’ sialylation while preserving systemic glycosylation. Although podocin promoter–driven Cre expression begins during early glomerular development, loss of sialylation becomes evident only around postnatal day 21, followed by proteinuria and progressive renal dysfunction. Time-course analyses demonstrated that podocyte desialylation triggered foot process effacement and cellular stress, followed by podocyte detachment and glomerular sclerosis [20].

At the molecular level, loss of sialylation affects the normal distribution of nephrin, a key slit diaphragm protein. In healthy conditions, sialylated nephrin presents a linear capillary loop pattern, whereas desialylated nephrin accumulates in intracellular vesicles and is reduced in surface expression, consistent with impaired trafficking and altered protein turnover [20,23].

In vitro, sialylation-deficient podocytes retain the ability to proliferate and differentiate but fail to grow out from isolated glomeruli and present reduced adhesion to collagen, a major component of the glomerular basement membrane [20]. These findings indicate a key role of sialylation in podocyte-matrix interactions rather than in basic cell viability.

Overall, sialylation emerges as a critical determinant of podocyte structural integrity and slit loss may represent an early and potentially reversible step in glomerular disease progression.

### 3.2. Sialic Acid and Glomerulopathies

Evidence from both renal and non-renal diseases highlights the dual role of sialic acids as biomarkers and modulators of immune and inflammatory processes. A common mechanism is the dysregulation of sialyltransferases and sialidases, which alters the distribution of sialic acids on cell membranes and circulating molecules. In the kidney, these changes affect immune recognition, complement regulation, and glomerular integrity, particularly in immune-mediated glomerulopathies [5,10,16].

In IgA nephropathy (IgAN), although galactose-deficient O-glycans are central to disease pathogenesis, abnormal N-glycosylation of IgA has been shown to contribute to disease progression [24]. Changes in IgAN-glycan sialylation promote the formation of polymeric IgA and favor immune complex deposition in the mesangium. Moreover, altered sialylation of both IgA and its receptor FcαR modulates binding affinity, influencing mesangial activation and immune complex clearance. In IgAN associated with alcoholic cirrhosis, a secondary form of glomerular disease, impaired hepatic clearance of sialylated N-glycans further alters IgA1 glycosylation and is associated with enhanced transferrin receptor expression and glomerular deposition [5,14,24,25].

Dysregulated sialic acid–containing N-glycans are also implicated in other immune-mediated glomerulopathies. In anti–glomerular basement membrane disease, aberrant N-glycosylation of myeloperoxidase, including altered site occupancy and glycan truncation, has been shown to expose neo-epitopes targeted by pathogenic autoantibodies, thereby driving immune complex–mediated glomerular injury. In primary membranous nephropathy, abnormal IgG4 N-glycosylation, characterized by reduced galactosylation and altered sialylation, has been associated with complement activation and podocyte damage. Notably, antibody deglycosylation abolishes pathogenicity, underscoring the functional relevance of glycan-dependent immune mechanisms [5,14].

Complement-mediated glomerulopathies such as C3 glomerulopathy (C3G) may suggest an example of how altered sialic acid–dependent complement regulation may contribute to disease pathogenesis. C3G is characterized by abnormal activation of the alternative complement pathway, often driven by genetic or acquired defects affecting complement regulators such as factor H. Given the central role of sialic acids in mediating factor H binding to host surfaces, alterations in sialylation are likely to impair complement control at the glomerular level, favoring persistent C3 activation and deposition. However, direct mechanistic evidence remains limited, and disruption of sialic acid–dependent complement regulation should be considered a plausible but not yet fully established mechanism contributing to complement-mediated injury in C3G [5,15,26,27].

In lupus nephritis, sialic acid metabolism has been shown to modulate glomerular inflammation through multiple pathways. Neuraminidase-mediated desialylation of N-glycans unmasks pattern-recognition receptors such as TLR4 on mesangial cells, triggering p38 and ERK/MAPK signaling and promoting pro-inflammatory cytokine release. Concurrently, shifts in IgG N-glycan sialylation and fucosylation regulate podocyte-injuring pathways, including CaMK4 expression. These mechanisms integrate innate and adaptive immune responses, amplifying local inflammation and tissue damage [5,14,28,29,30].

Across these conditions, loss, modification, or redistribution of sialic acid–containing glycans enhances immune recognition, promotes complement activation, and sustains inflammatory signaling. Altered sialylation affects circulating immune complexes and reshapes cell–cell interactions, promoting mesangial activation, leukocyte recruitment, and podocyte injury. Overall, sialic acid–dependent mechanisms emerge as a unifying framework linking immune dysregulation, complement activation, and progressive glomerular damage.

### 3.3. Sialic Acid in Kidney Transplantation

Among glycosylated molecules, immunoglobulin G (IgG) represents a particularly relevant example, as its Fc region carries a conserved N-linked glycan that can modulate antibody effector functions. Variations in Fc glycosylation have been associated with altered interactions with Fc receptors and complement components, thereby influencing the pro- or anti-inflammatory activity of antibodies [31,32,33,34].

In kidney transplantation, IgG antibodies directed against donor human leukocyte antigens (HLA), commonly referred to as donor-specific antibodies (DSA), play a central role in antibody-mediated rejection (AMR), one of the leading causes of graft dysfunction and loss. DSA can trigger complement activation, endothelial injury, and microvascular inflammation, ultimately compromising graft survival [31,33,35,36].

However, clinical observations indicate that the mere presence or titer of DSA does not fully predict clinical outcome. At comparable DSA levels, some patients develop severe rejection, whereas others remain clinically stable. This variability suggests that additional qualitative features of antibodies contribute to their pathogenic potential. In this context, growing attention has been directed toward IgG glycosylation as a potential determinant of DSA function and pathogenicity.

The study by Barba T. et al. investigated whether the degree of sialylation of DSA influences the severity of AMR. In a cohort of 69 patients with histologically confirmed AMR, DSA were identified using HLA single-antigen bead assays, and their sialylation was assessed by lectin-based ELISA. Despite substantial inter-individual variability, sialylation levels were not associated with histological severity, complement activation, or graft survival, suggesting that sialylation alone does not determine clinical outcome [31].

A more integrative perspective was provided by Noble J. et al. (2025) [33], which analyzed 65 transplant recipients stratified by rejection status (no rejection, acute AMR, chronic AMR): using a panel of lectin-based assays to characterize multiple Fc glycan features, including sialylation, fucosylation, mannose content, and bisecting GlcNAc, the study showed that sialylation is not a major driver of rejection severity. Instead, other glycosylation traits, particularly increased bisecting GlcNAc, were associated with microvascular inflammation and chronic injury, supporting the idea that IgG function is shaped by a combination of glycan features [33]. However, as the study has not yet been peer-reviewed, these findings should be interpreted with appropriate caution.

Overall, these findings support the notion that IgG effector function is not governed by a single modification but rather by a combinatorial “glycan code”. In this context, emerging approaches such as graft glyco-engineering, illustrated by a study on short-term enzymatic removal of ABO antigens during machine perfusion, offer an additional perspective on how modulation of glycosylation may influence alloimmune responses. While operating at the level of the graft rather than circulating antibodies, these strategies further highlight the complexity of glycan-mediated regulation, suggesting that the impact of individual modifications, including sialylation, is likely to depend on their broader biological context [32].

### 3.4. Sialic Acid and Plasma Cell Dyscrasias (Multiple Myeloma)

Plasma cell dyscrasias, including multiple myeloma, are characterized by the production of monoclonal immunoglobulins with distinct structural and functional properties that can profoundly affect both systemic immunity and renal function. In this context, alterations in protein glycosylation, and particularly sialylation, have emerged as key modulators of tumor–immune interactions and disease pathophysiology [37,38,39,40].

A hallmark of multiple myeloma is the presence of a hypersialylated tumor cell surface, resulting from dysregulated sialyltransferase activity. This altered glycan landscape is not merely structural but functionally active, contributing to tumor progression, cell trafficking, and resistance to therapy. From a mechanistic perspective, hypersialylation enables malignant plasma cells to exploit sialic acid–dependent immune checkpoint pathways [38,39,40].

A central mechanism involves the interaction between tumor-associated sialylated glycans and inhibitory SIGLEC receptors expressed on immune cells, particularly natural killer (NK) cells [38]. Myeloma cells express high levels of ligands for SIGLEC-7 and SIGLEC-9, including glycoproteins such as PSGL-1, which function as dominant binding partners. Engagement of these ligands with SIGLEC-7 on NK cells triggers intracellular inhibitory signaling through ITIM domains, leading to recruitment of phosphatases such as SHP-1 and SHP-2. This results in attenuation of NK cell activation, reduced degranulation, and impaired cytotoxicity. In this way, hypersialylation effectively establishes a glyco-immune checkpoint, allowing tumor cells to evade immune surveillance [37,38,41,42].

Importantly, experimental desialylation of myeloma cells, either through enzymatic removal of sialic acids or inhibition of sialyltransferases, restores NK cell function. This effect is mediated by disruption of SIGLEC–ligand interactions and is associated with increased NK cell degranulation, cytokine production (including IFN-γ and TNF-α), and tumor cell lysis. Moreover, genetic deletion of SIGLEC-7 in NK cells further enhances cytotoxic responses, confirming the central role of this axis in immune escape [38,42].

Beyond immune checkpoint signaling, sialylation also influences antigen accessibility and antibody-based therapies. Hypersialylation can sterically mask surface antigens such as CD38, a key therapeutic target in multiple myeloma. Desialylation has been shown to increase CD38 detectability on tumor cells, thereby enhancing antibody-dependent cellular cytotoxicity (ADCC) induced by anti-CD38 monoclonal antibodies. This dual effect, the removal of inhibitory signaling and the unmasking of target antigens, suggests that sialylation operates at multiple mechanistic levels to regulate tumor–immune interactions [38,43,44,45].

In addition to these immune-mediated effects, altered glycosylation of monoclonal immunoglobulins and light chains may influence renal involvement in plasma cell dyscrasias. Although the role of sialylation in light chain nephrotoxicity remains incompletely defined, changes in glycan composition may affect protein folding, aggregation, and interactions with tubular epithelial cells, thereby modulating the development of cast nephropathy and other forms of monoclonal gammopathy–associated kidney injury [38].

These findings identify sialylation as a central regulator of tumor immune evasion, antibody function, and potentially renal toxicity in plasma cell dyscrasias. By integrating glycan-mediated immune checkpoints with structural and biochemical effects on immunoglobulins, the sialic acid axis represents a promising target for therapeutic intervention aimed at enhancing anti-tumor immunity and mitigating organ damage.

## 4. Therapeutic Perspectives

The growing recognition that altered sialylation contributes to both glomerular and tubulointerstitial injury has opened a new therapeutic landscape in kidney diseases. Sialic acids, as terminal negatively charged residues capping glycoproteins and glycolipids, play a fundamental role in maintaining the structural and electrostatic integrity of the glomerular filtration barrier while also regulating cell signaling, receptor accessibility, and extracellular matrix interactions. Accordingly, therapeutic strategies aimed at modulating sialic acid metabolism can be broadly categorized into two complementary approaches: (i) inhibition of excessive desialylation and (ii) restoration of defective sialic acid biosynthesis.

### 4.1. Inhibition of Pathological Desialylation: Targeting Sialidases

Human cells express four distinct exo-sialidases (neuraminidases), NEU1–NEU4, each capable of removing terminal sialic acid residues from glycoconjugates. Although these enzymes differ in subcellular localization, substrate specificity, and tissue distribution, their functions are only partially overlapping. NEU1 is the most abundantly expressed isoform in human tissues, whereas NEU2 shows relatively low expression; NEU3 and NEU4 display intermediate levels and have been increasingly implicated in pathological processes [46,47,48,49,50].

Sialidases fine-tune cellular behavior by modulating the sialylation status of membrane glycoproteins and gangliosides. Under physiological conditions, this activity regulates receptor availability and signal transduction. However, in disease states, excessive or dysregulated desialylation may become maladaptive, exposing receptor-binding domains, amplifying signaling pathways, and promoting inflammation and fibrosis. Indeed, aberrant sialidase activity has been linked to a broad spectrum of conditions, including fibrosis, cancer, metabolic disorders, and neurodevelopmental diseases. Importantly, multiple sialidase isoforms may contribute simultaneously to pathological remodeling, underscoring the need for isoform-specific therapeutic targeting [46,47].

Advances in structural biology, particularly crystallographic characterization of NEU2, have enabled homology modeling of other isoforms and facilitated structure-based drug design. Differences in sequence homology, notably the lower similarity of NEU1 compared to NEU2–4, have guided the development of selective inhibitors. Modified sialic acid analogs have shown promising isoform selectivity: C9 biphenyl carbamate derivatives preferentially inhibit NEU3, whereas amide and triazole derivatives demonstrate greater activity against NEU1 and NEU4, respectively. In addition, the ability of NEU1 and NEU3 to form dimers suggests that disruption of protein–protein interactions may represent an alternative inhibitory strategy [46,47,51].

Within the kidney, emerging evidence identifies NEU4 as a key driver of renal fibrosis. In experimental models such as unilateral ureteral obstruction and folic acid–induced injury, NEU4 is markedly upregulated in tubular epithelial cells, which are central mediators of fibrotic progression. Genetic deletion or silencing of NEU4 attenuates epithelial-to-mesenchymal transition (EMT), reduces extracellular matrix deposition, and limits inflammatory cell infiltration, whereas NEU4 overexpression exacerbates fibrotic remodeling [46,47].

NEU4 directly interfaces with intracellular signaling pathways through interaction with Yes-associated protein (YAP), a core effector of the Hippo pathway and a master regulator of mechanotransduction and fibrogenic gene expression. NEU4 stabilizes YAP and promotes its nuclear translocation, thereby enhancing transcription of pro-fibrotic genes. Conversely, NEU4 inhibition increases YAP phosphorylation, favoring cytoplasmic retention and proteasomal degradation. This NEU4–YAP axis provides a direct molecular link between altered sialylation and transcriptional programs driving renal fibrosis [47,52,53,54,55].

Pharmacological targeting of NEU4 has yielded encouraging preclinical results. Screening of natural compounds identified 3,5,6,7,8,3′,4′-heptamethoxyflavone (HMF) as a selective NEU4 inhibitor. HMF disrupts the NEU4–YAP interaction and significantly attenuates kidney fibrosis in murine models; however, isoform selectivity and systemic safety, particularly regarding potential effects in non-renal tissues, remain to be established [47]. These findings position NEU4 inhibition as a promising anti-fibrotic strategy, complementing established approaches targeting TGF-β and related pathways [52,53,54,55].

In addition, other sialidase isoforms have been implicated in renal pathophysiology, although the available evidence remains more limited. NEU1 has been associated with fibrotic progression in murine models of lupus nephritis, where its modulation, either through genetic knockout or adeno-associated virus–mediated approaches, affects disease severity and extracellular matrix remodeling. By contrast, NEU3 has been primarily linked to renal malignancies rather than fibrotic disease, with increased expression reported in cellular models of human renal cell carcinoma [56,57,58,59]. These findings suggest that, while NEU4 appears to play a central role in renal fibrosis, other isoforms may also contribute to kidney pathology in a manner dependent on the specific pathological context, highlighting the need for further investigation.

### 4.2. Restoring Sialic Acid Biosynthesis

In contrast to strategies aimed at limiting desialylation, a second therapeutic approach focuses on restoring insufficient sialylation. Sialic acid biosynthesis depends on the bifunctional enzyme UDP-GlcNAc 2-epimerase/N-acetylmannosamine kinase (GNE/MNK), which catalyzes the rate-limiting steps of the pathway. Experimental mouse models carrying the M712T mutation in the *Gne* gene have provided compelling evidence that systemic hyposialylation directly induces glomerular injury [51].

In these models, defective sialic acid synthesis leads to early podocyte foot process effacement, glomerular basement membrane abnormalities, and severe proteinuria. Key glomerular proteins, including podocalyxin and nephrin, exhibit biochemical evidence of hyposialylation. Given the central role of the negatively charged glycocalyx in maintaining charge selectivity, these findings establish impaired sialylation as a primary determinant of filtration barrier dysfunction [51,60,61].

Importantly, supplementation with N-acetylmannosamine (ManNAc), a metabolic precursor downstream of the defective enzymatic step, partially rescues this phenotype. By bypassing the defective kinase activity, ManNAc restores intracellular sialic acid production. Treated animals show improved podocyte architecture, reduced GBM alterations, normalization of lectin-binding profiles, and decreased albuminuria. These results provide proof-of-concept that glomerular injury secondary to hyposialylation is, at least in part, reversible through metabolic intervention [51,60,61].

### 4.3. Clinical Translation: First-in-Human Evaluation of ManNAc

The translational potential of metabolic sialic acid restoration has recently been explored in a Phase 1 clinical trial of oral ManNAc in adults with primary podocytopathies, including focal segmental glomerulosclerosis, minimal change disease, and membranous nephropathy. These conditions are characterized by podocyte dysfunction and significant proteinuria, and accumulating evidence suggests that altered sialylation contributes to disease pathogenesis in a subset of patients [62].

The study demonstrated that oral ManNAc is safe and well-tolerated across ascending dose regimens. Pharmacokinetic analyses revealed rapid absorption and measurable increases in plasma N-acetylneuraminic acid (Neu5Ac) within hours of administration. As expected, reduced renal function influenced systemic exposure, highlighting the need for dose optimization in advanced chronic kidney disease [62].

Although not powered to assess efficacy, the trial reported early reductions in proteinuria, ranging from modest to substantial across individuals. Notably, the magnitude of response appeared to correlate with baseline glomerular hyposialylation, supporting a mechanism-based therapeutic effect [62]. These findings suggest that enhancement of glomerular sialylation may restore filtration barrier integrity in selected patient populations.

Ongoing Phase 2 studies will be critical to define long-term efficacy, optimal dosing strategies, and patient stratification. If confirmed, ManNAc supplementation could represent the first metabolism-based therapeutic approach targeting glomerular diseases driven by defective sialylation.

## 5. Conclusions

Sialic acids emerge as central regulators of kidney disease, linking immune activation, complement control, and glomerular injury. Altered sialylation affects key pathogenic pathways, including immune recognition, complement activation, and podocyte integrity, thereby contributing to both inflammation and fibrosis.

Across glomerulopathies, transplantation, and plasma cell dyscrasias, changes in glycan composition consistently modulate disease expression and progression, supporting the concept that sialylation is not only a biomarker of disease activity but also a functional determinant of pathogenic mechanisms.

Importantly, targeting sialic acid metabolism represents a promising therapeutic strategy, as both inhibition of pathological desialylation and restoration of sialylation pathways may modify disease course. Overall, sialylation can be viewed as (i) a critical regulator of immune and complement-mediated kidney injury, (ii) a contributor to disease progression across diverse renal conditions, and (iii) a novel, mechanism-based therapeutic target.

Overall, emerging clinical and preclinical data support a relevant and potentially broader role of sialic acid biology and protein sialylation in kidney physiology and disease. Growing evidence also suggests that sialylation-dependent mechanisms may represent a general regulatory layer across multiple tissues, further supporting the translational relevance of this pathway.

## Data Availability

No new data were created or analyzed in this study. Data sharing is not applicable to this article.
